# Three-dimensional deep learning nomogram for post-plan quality assurance in high-dose-rate prostate brachytherapy^☆^

**DOI:** 10.1016/j.phro.2026.101100

**Published:** 2026-10-02

**Authors:** Joel Beaudry, Tonghe Wang, Shirin A. Enger, Antonio L. Damato

**Affiliations:** a Department of Medical Physics, Memorial Sloan Kettering Cancer Center, New York, NY, USA; b Medical Physics Unit, Department of Oncology, McGill University, Montreal, Quebec, Canada; c Lady Davis Institute for Medical Research, Jewish General Hospital, Montreal, Quebec, Canada

**Keywords:** High-dose-rate brachytherapy, Prostate cancer, Quality assurance, Deep learning, Nomogram, Isodose volume verification

## Abstract

**Purpose:**

Classical nomograms for high-dose-rate (HDR) prostate brachytherapy quality assurance (QA) evaluate agreement between planned and expected total dwell time but do not directly assess the three-dimensional treated volume. This study's aim was to develop a deep learning-based 3D nomogram to predict institution-specific clinical 100% isodose volumes from anatomy and dwell positions for post-plan QA.

**Methods:**

A total of 2394 single-fraction 15 Gy HDR prostate brachytherapy plans were included. Input label maps encoded the 3D prostate, urethra, rectum, and dwell positions. The prediction target was the clinical 100% isodose volume. A five-fold cross-validation strategy was used for model development, with 2155 plans used for training and validation and 239 plans held out for testing. A 3D U-Net was trained using combined Dice and binary cross-entropy loss. Agreement was evaluated using prostate V100%, 100% isodose volume, Dice similarity coefficient, and average symmetric surface distance (ASSD). Validation-derived thresholds were used for outlier screening and targeted review.

**Results:**

The model predicted prostate V100% with a mean error of 0.73 percentage points. Predicted and clinical 100% isodose volumes showed strong agreement, with R^2^ = 0.990, slope = 0.99, and intercept = 0.77 cm^3^. Median Dice similarity coefficient was 0.960, and median ASSD was 0.48 mm. A 95% threshold flagged 6.3% of plans as atypical relative to validation data.

**Conclusions:**

A deep learning-based nomogram predicted an institution-specific 100% isodose volume with low coverage error and high spatial agreement. This geometry aware post-plan QA tool can complement equation-based nomograms by directly evaluating the spatial treated volume.

## Highlights

• Deep learning predicted three-dimensional 100% isodose volume for high-dose-rate prostate plans.
• Median prostate V_100%_ error was 0.73 percentage points.
• Predicted and clinical treated 100% isodose volumes had a median Dice of 0.960.
• Geometry-aware quality assurance identified plans deviating from historical patterns.

## Graphical abstract

**Figure:**
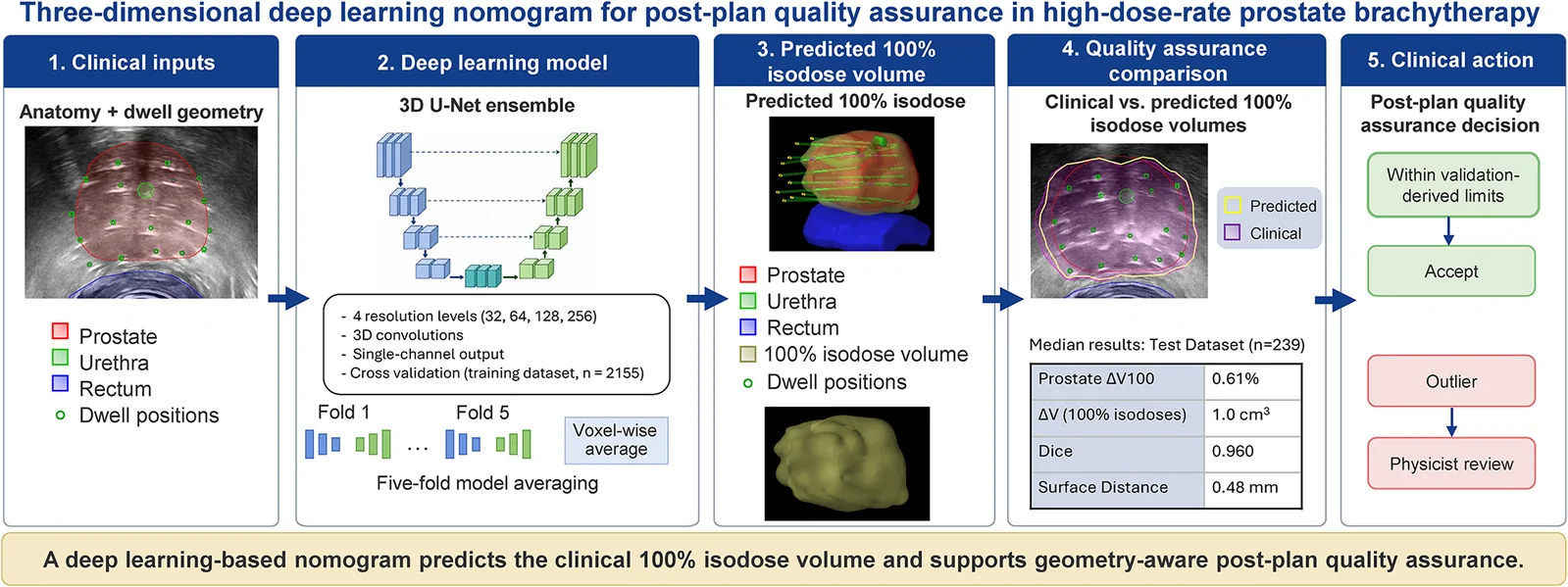

## 1 Introduction

High-dose-rate (HDR) prostate brachytherapy planning aims to achieve high target coverage while respecting urethral and rectal dose constraints. In routine practice, rapid independent checks verify completed-plan plausibility before treatment delivery [1]. Classical prostate brachytherapy nomograms provide one such method by relating scalar plan descriptors, such as implant volume or the 100% isodose volume, to expected plan outputs such as total dwell time [2], [3]. Equation-based nomograms use the clinical 100% isodose volume ($TV_{100\%}^{\mathrm{clin}}$, cm^3^) to predict the total dwell time for comparison with the clinically planned value [4]. Deviations greater than 10% prompt additional review by a physicist and documentation.

Several studies have demonstrated the use of nomograms and simple analytical models as efficient secondary checks for HDR prostate brachytherapy plans [3], [5]. Equation-based nomograms can find relationships between many variables: target volume and total reference air kerma, total irradiation time and volume, and, dose-volume fits checking integral dwell times with known isodose volumes [6], [7], [8].

Despite their clinical utility, equation-based nomograms have intrinsic limitations. Even when the 100% isodose volume is used to reflect peripheral dwell geometry, scalar summaries collapse a three-dimensional implant into a single number and cannot localize spatial discrepancies. The relationship between volume-based descriptors and dwell time also depends on local implant technique and planning strategy, and may require recalibration over time [3]. Technically challenging cases, such as very small glands, unusual anatomy, or elongated targets, can deviate from the assumptions of simple regression models and may be precisely where an independent plausibility check is most valuable [9].

Deep learning has been explored for three-dimensional dose prediction and dose calculation in prostate and cervical brachytherapy [10], [11]. However, a spatial prediction framework combining anatomy and dwell geometry for HDR prostate post-plan quality assurance (QA) and benchmarked against a clinical equation-based nomogram has not been evaluated. We therefore developed and evaluated a three-dimensional U-Net that predicted the clinical 100% prescription isodose volume as a binary mask from prostate, urethra, rectum, and dwell-location labels. Using five-fold cross-validation and a held-out test set, we quantified coverage, volume, and spatial agreement, derived validation-based outlier thresholds for physicist review, and compared the spatial model with a clinical equation-based dwell-time nomogram.

## 2 Material and methods

### 2.1 Study design and clinical workflow

This retrospective study evaluated a three-dimensional deep learning nomogram for HDR prostate brachytherapy plan verification. From anatomical contours (prostate, urethra, and rectum) and planned dwell locations in each approved plan, the model predicted the 100% isodose volume ($TV_{100\%}^{\mathrm{pred}}$) as a binary mask for comparison with the clinical volume ($TV_{100\%}^{\mathrm{clin}}$). The workflow is shown in Fig. 1.

**Fig. 1:**
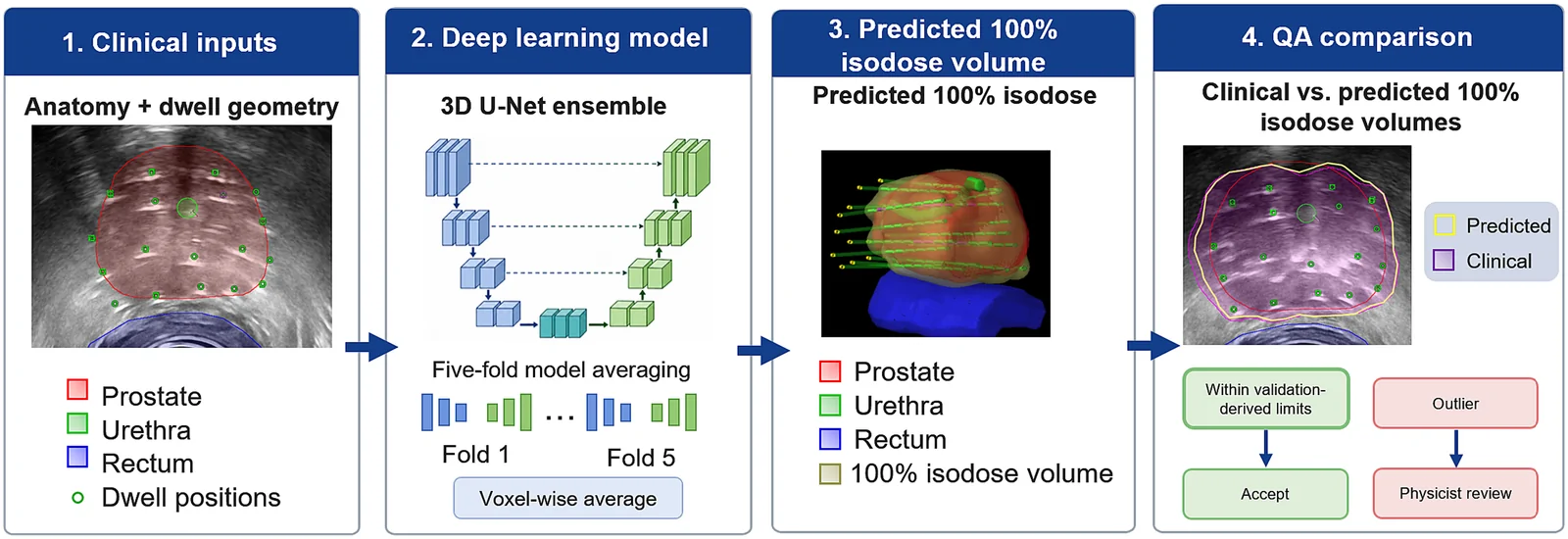
Overview of the deep-learning nomogram QA pipeline. A single 3D label-map input encodes prostate, urethra, rectum, and dwell locations. The model outputs a 3D predicted 100% isodose mask, ${\mathrm{TV}}_{100\%}^{\mathrm{pred}}$, which is compared against the $TV_{100\%}^{\mathrm{clin}}$ to compute (i) prostate V_100%_ coverage agreement and (ii) spatial agreement metrics (Dice, ASSD, HD_99_), enabling plan QA verification.

All cases represent HDR prostate brachytherapy planned to deliver 15 Gy in a single fraction, with external beam radiotherapy delivered subsequently per institutional practice [12]. All included cases were whole-gland plans, and no focal treatment plans were included. Planning objectives followed institutional protocol consistent with American Brachytherapy Society guidance, including prostate V_100%_ ≥ 95%, urethra D_max_ ≤ 120% and D_10%_ ≤ 110%, and rectum D_max_ ≤ 100% with $D_{2cm^{3}}$ ≤ 70% and $D_{1cm^{3}}$ ≤ 80% [13]. Dose-volume notation follows standard convention; V_xx%_ denotes the fraction of a structure receiving at least xx% of prescription dose. Predicted and clinical 100% isodose volumes were compared using volumetric and spatial agreement measures. Prostate coverage was summarized using prostate V_100%_. The study cohort comprised plans generated between 2018 and 2025. During this period, clinical planning involved 19 medical physicists and 7 radiation oncologists.

### 2.2 Data and representations

A single-institution retrospective dataset of HDR prostate brachytherapy plans was used (*n* = 2394). The study was approved by the institutional review board (protocol no. 16–660). Splitting was performed at the patient level, with each patient contributing one single-fraction plan to the dataset. A held-out test set (*n* = 239) was excluded from model development. The remaining cohort (*n* = 2155) was used for model development and evaluated using five-fold cross-validation. The five fold-specific models were subsequently used for ensemble inference on the held-out test set.

For each plan, the input volume was a single-channel 3D integer label map derived from physician-drawn contours and planned dwell locations. Dwell times were not included in the model input. Contours, dose distributions, and dwell coordinates were exported from Vitesse (Varian Medical Systems, Palo Alto, CA, USA) in the same Digital Imaging and Communications in Medicine (DICOM) coordinate frame. Contours and dose-derived masks were converted to the image grid using nearest-neighbour interpolation to preserve binary labels. Dwell coordinates were extracted from the RT Plan DICOM file and rasterized onto the image grid. All possible dwell positions along each reconstructed catheter were included. The label encoding was 0 = background, 1 = prostate, 2 = urethra, 3 = rectum, and 4 = dwell locations. To preserve visibility after network downsampling, dwell locations were dilated using a spherical structuring element with a radius of 1 mm.

The target was a three-dimensional binary mask representing the clinical 100% isodose volume ($TV_{100\%}^{\mathrm{clin}}$), generated from the treatment planning system dose distribution by thresholding at 100% of the prescription dose. All input label maps and target masks were resampled to a common voxel spacing of (dz, dy, dx) = (1.0, 0.5, 0.5) mm. Volumes were cropped 80 × 128 × 128 voxels to remove non-relevant regions and standardize input dimensions across cases.

### 2.3 Deep learning model

A three-dimensional MONAI-based 3D U-Net architecture was used [14], [15]. The network consisted of four resolution levels with convolutional channel sizes of 32, 64, 128, and 256, with three stride-2 downsampling stages applied in each spatial direction. Models were trained independently within each fold using AdamW (initial learning rate 1 × 10^−4^) and an equally weighted Dice plus binary cross-entropy loss. Training used validation Dice for learning-rate scheduling and early stopping, with a maximum of 200 epochs and batch size 4. On-the-fly MONAI spatial augmentation included random X/Z flips and random affine translations up to 10 mm [15]. At test time, the five-fold specific models were combined as an ensemble by averaging per-voxel sigmoid probabilities and thresholding at 0.5 to produce the predicted 100% isodose volume mask (${\mathrm{TV}}_{100\%}^{\mathrm{pred}}$). To improve transparency and reproducibility, a structured AID-RT model card is provided in Supplementary Material A [16].

### 2.4 Evaluation and comparison

The primary endpoint was prostate coverage, summarized as prostate V_100%_ and computed as the fraction of prostate voxels within the predicted 100% isodose volume mask:

(1) $$V_{100\%}^{\mathrm{pred}}=100\;x\;\left|\;{\mathrm{TV}}_{100\%}^{\mathrm{pred}}\cap \mathrm{Prostate}\;\right|/|\;\mathrm{Prostate}|$$

A secondary volumetric endpoint was the 100% isodose volume, ${\mathrm{TV}}_{100\%}$ (cm^3^). Spatial agreement between ${\mathrm{TV}}_{100\%}^{\mathrm{pred}}$ and $TV_{100\%}^{\mathrm{clin}}$ was evaluated using the Dice similarity coefficient (DSC), average symmetric surface distance (ASSD), and 99th percentile Hausdorff distance (HD_99_).

For each case, signed coverage error was defined as $\Delta V_{100\%}=V_{100\%}^{\mathrm{pred}}$ - $V_{100\%}^{\mathrm{clin}}$ and reported in percentage points. A validation-derived calibration offset was defined as the mean $\Delta V_{100\%}$ across out-of-fold validation predictions. Warning and action thresholds were then defined as the 90th and 95th percentiles of the bias-centered absolute error, |$\Delta V_{100\%}$- bias|. Analogous percentile thresholds were derived for ${\mathrm{TV}}_{100\%}$spatial agreement metrics, DSC and ASSD. Because the held-out test set consisted of clinically approved plans without independent error labels, these validation-derived thresholds are intended for outlier screening or flagging cases for review not error-detection accuracy. Bland–Altman analysis was used separately to summarize systematic bias and 95% limits of agreement between predicted and clinical metrics.

A conventional equation-based nomogram was used in comparison, as implemented clinically for independent plan verification. The nomogram predicts total treatment time per unit prescription dose as:

(2) $$\begin{array}{c} T\cdot Gy^{-1}=2.247\cdot {{\mathrm{IV}}_{100\%}^{2}}^{\left(2/3\right)}\cdot \exp\left(0.07\cdot \left(E-1\right)\right). \end{array}$$

Here, T is total treatment time (s), IV_100%_ is the 100% isodose volume (cm^3^), and E is an elongation term defined as the ratio of the long to short implant dimension (2). For a 15 Gy prescription, the predicted total time is *T* = 15 Gy x (T·Gy^−1^). Nomogram-predicted times were obtained from the institutional workflow and compared with clinically planned total dwell times.

Statistical reporting included least-squares regression, error summaries, Bland–Altman bias and 95% limits of agreement, and residual analysis. Residuals were defined as $TV_{100\%}^{\mathrm{pred}}$ - $TV_{100\%}^{\mathrm{clin}}$ (cm^3^) for the deep learning model and predicted minus clinical total dwell time (s) for the classical nomogram.

### 2.5 Sensitivity testing

Qualitative label-map perturbations assessed whether model output responded coherently to plausible changes in anatomy and dwell geometry in one representative test case. Perturbations included prostate expansion, removal of dwell positions from one anterior needle, and urethra removal by relabeling its voxels as prostate. These tests were intended as qualitative check of model behavior under plausible deviations in anatomy and dwell geometry.

## 3 Results

On the test set, the mean absolute error in prostate V_100%_ was 0.94 percentage points. The mean and median signed error was +0.73 and + 0.61 percentage points, respectively. Overall, 88.3% of cases were within ±2 percentage points and 99.6% were within ±5 percentage points. Fig. 2 shows the distribution of $\Delta V_{100\%}$.

**Fig. 2:**
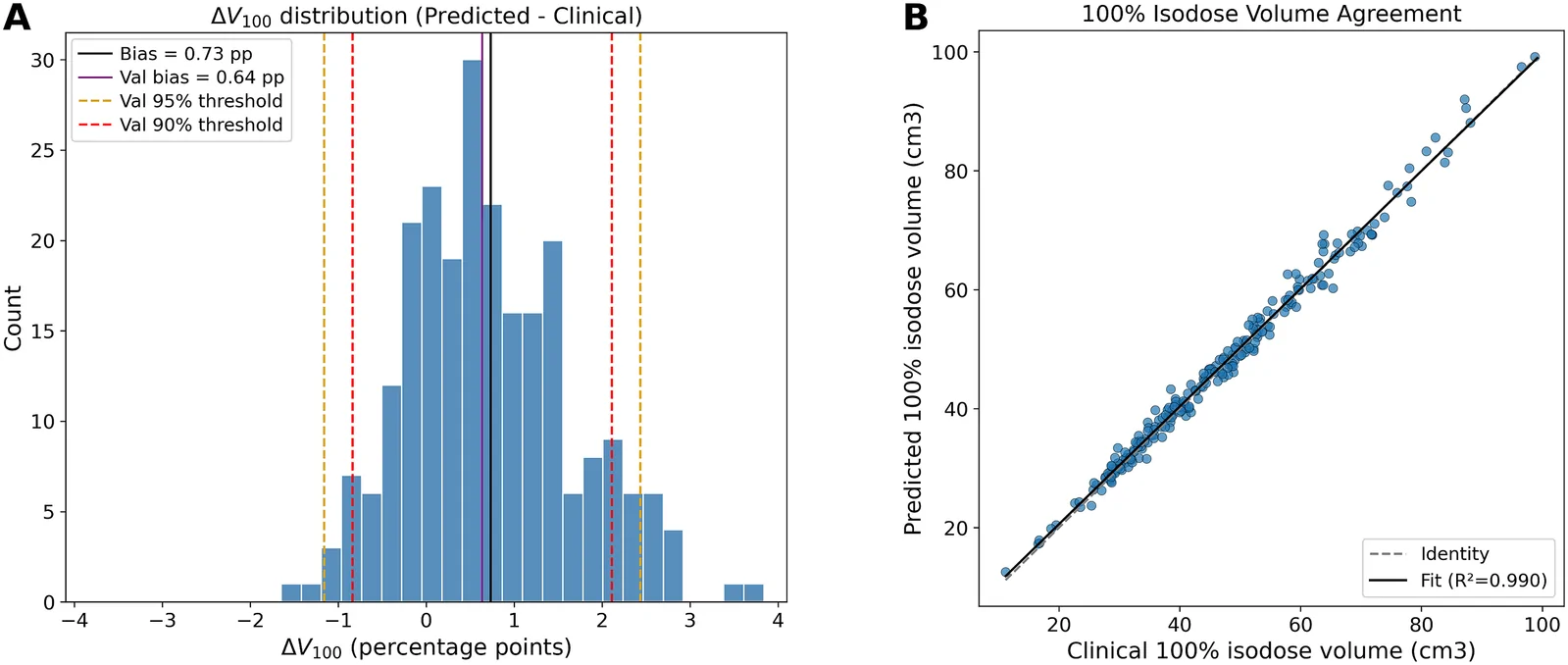
(A) Distribution of prostate coverage error, $\Delta V_{100\%}$, with reference lines at ±2 and ± 5 percentage points and validation-derived thresholds overlaid. Vertical lines indicate the observed test-set bias, the validation-derived bias (calibration offset), and validation-derived containment thresholds for outlier screening (90% and 95%). (B) $V_{100\%}^{\mathrm{pred}}$ versus $TV_{100\%}^{\mathrm{clin}}$, with identity line and least-squares fit. (*n* = 239).

Volumes of ${\mathrm{TV}}_{100\%}^{\mathrm{pred}}$ and $TV_{100\%}^{\mathrm{clin}}$ showed agreement (R^2^ = 0.99, slope = 0.99, intercept = 0.77 cm^3^). The median absolute volume difference was 1.04 cm^3^ (IQR: 0.49 to 1.75 cm^3^), corresponding to a median absolute percentage difference of 2.5% relative to $TV_{100\%}^{\mathrm{clin}}$. Bland-Altman analysis showed a bias of +0.30 cm^3^ (95% CI, 0.09 to 0.50 cm^3^) with 95% limits of agreement from −2.86 to +3.46 cm^3^. Fig. 2 shows volumes of $TV_{100\%}^{\mathrm{pred}}$ versus $TV_{100\%}^{\mathrm{clin}}$, and the corresponding Bland-Altman analysis is shown in Fig. 3.

**Fig. 3:**
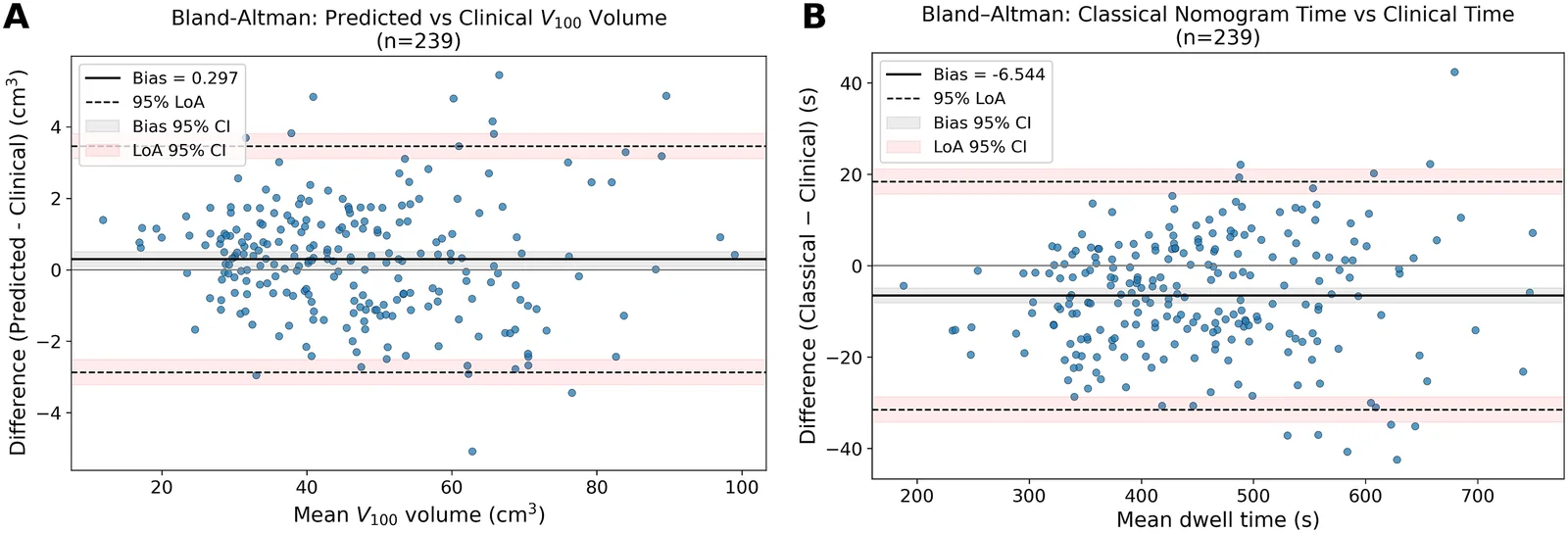
(A) Bland–Altman plot for ${\mathrm{TV}}_{100\%}^{\mathrm{pred}}$ and $TV_{100\%}^{\mathrm{clin}}$, with mean TV_100%_ volume (cm^**3**^) on the x-axis. (B) Bland–Altman plot for classical nomogram predicted total time and clinically planned total time (seconds). Both plots display bias and 95% limits of agreement with confidence intervals (*n* = 239).

Spatial overlap and surface distance metrics between ${\mathrm{TV}}_{100\%}^{\mathrm{pred}}$ and $TV_{100\%}^{\mathrm{clin}}$ demonstrated high agreement. The median Dice similarity coefficient was 0.960 (IQR 0.953 to 0.964), and all cases achieved Dice ≥ 0.90. Surface distance metrics were low: median ASSD was 0.48 mm (IQR 0.42 to 0.56 mm), and median HD_99_ was 1.00 mm (IQR 0.50 to 1.00). Representative examples of agreement and disagreement between predicted and clinical 100% isodose volumes are shown in Fig. 4 corresponding Bland-Altman plot is show.

**Fig. 4:**
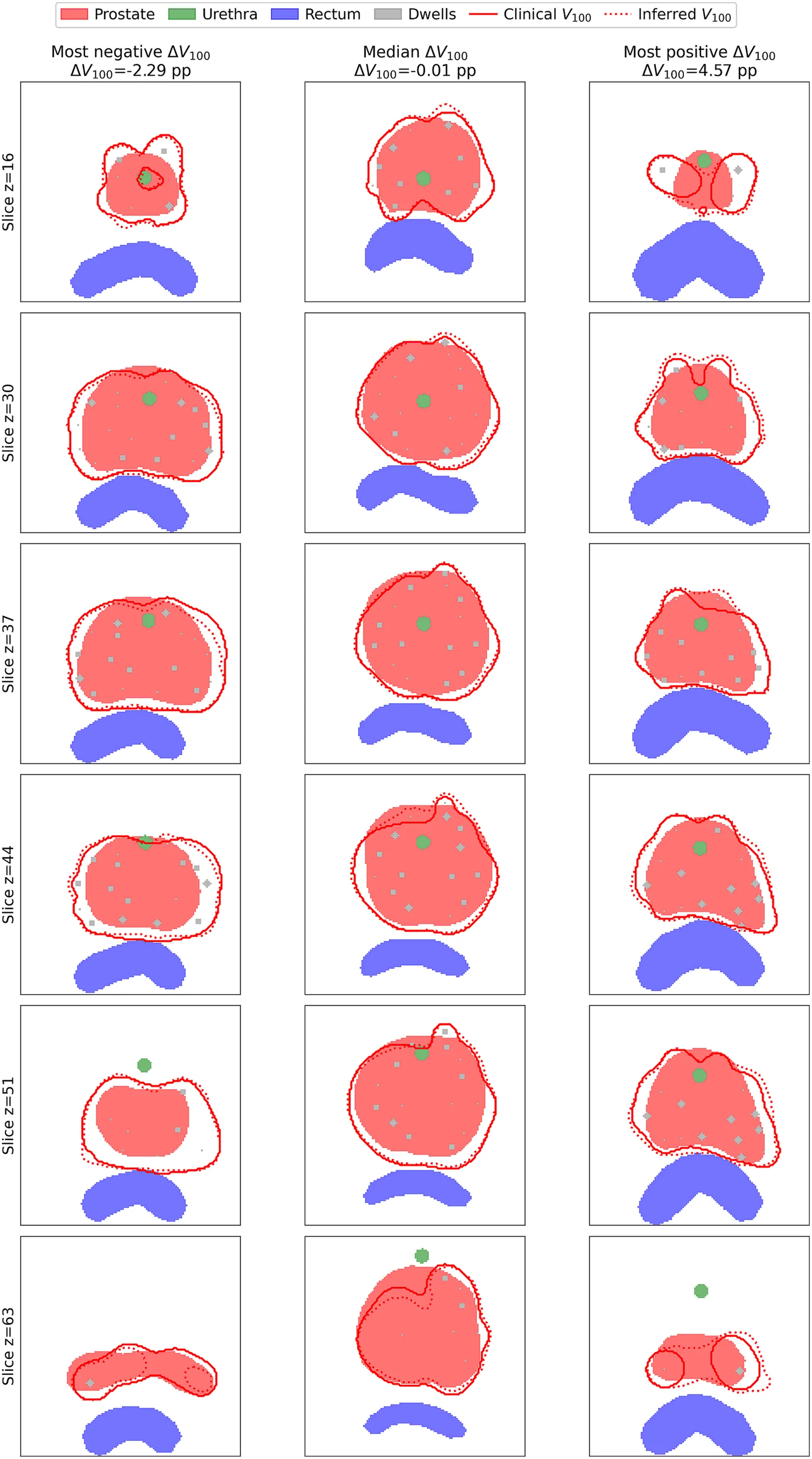
Representative axial slices from three test cases selected by bias-corrected ΔV_100%_ (most negative, median, most positive). Organ labels are shown as a colour overlay: prostate (red), urethra (green), rectum (blue), and dwells (gray). $TV_{100\%}^{\mathrm{clin}}$ is shown as a solid red line, and ***T***$V_{100\%}^{\mathrm{pred}}$ as a dotted red line. Six axial slices sampled along the cranio-caudal extent are displayed. (For interpretation of the references to colour in this figure legend, the reader is referred to the web version of this article.)

Validation predictions estimated prostate coverage bias and defined outlier-screening thresholds. Mean validation coverage bias was +0.638 percentage points. Containment-based thresholds provided two operating points: a 90% threshold of 1.47 percentage points flagged 13.4% of plans, and a 95% threshold of 1.80 percentage points flagged 6.3% as atypical. Flagged plans should be interpreted as prioritization for review, not evidence that the plans were incorrect or clinically unacceptable.

Similarly, spatial thresholds derived from validation were applied to the test set. At the 95% level, thresholds were Dice ≤0.938 (2.9% of cases exceeded) and ASSD ≥0.733 mm (3.8% of cases exceeded). For the combined QA scheme, Dice and ASSD were used as complementary spatial metrics alongside coverage agreement.

The classical equation-based nomogram showed agreement with clinical total dwell time (R^2^ = 0.985, slope = 0.984, intercept = 13.88 s). The mean absolute error was 11.25 s, corresponding to a mean absolute percentage error of 2.52% relative to clinical dwell time (SD 1.91%). Bland-Altman analysis (classical - clinical) showed a bias of −6.54 s (95% CI -8.17 to −4.92 s) with 95% limits of agreement from −31.51 to +18.43 s. The corresponding Bland-Altman plot is shown in Fig. 3.

Residuals for both methods are shown as a function of the corresponding clinical values in Fig. 5. For the deep learning-based model, ${\mathrm{TV}}_{100\%}$ residuals remained centered across the range of ${\mathrm{TV}}_{100\%}^{\mathrm{clin}}$ values. For the classical nomogram, dwell-time residuals were also centered but showed a broader spread across the range of isodose volumes.

**Fig. 5:**
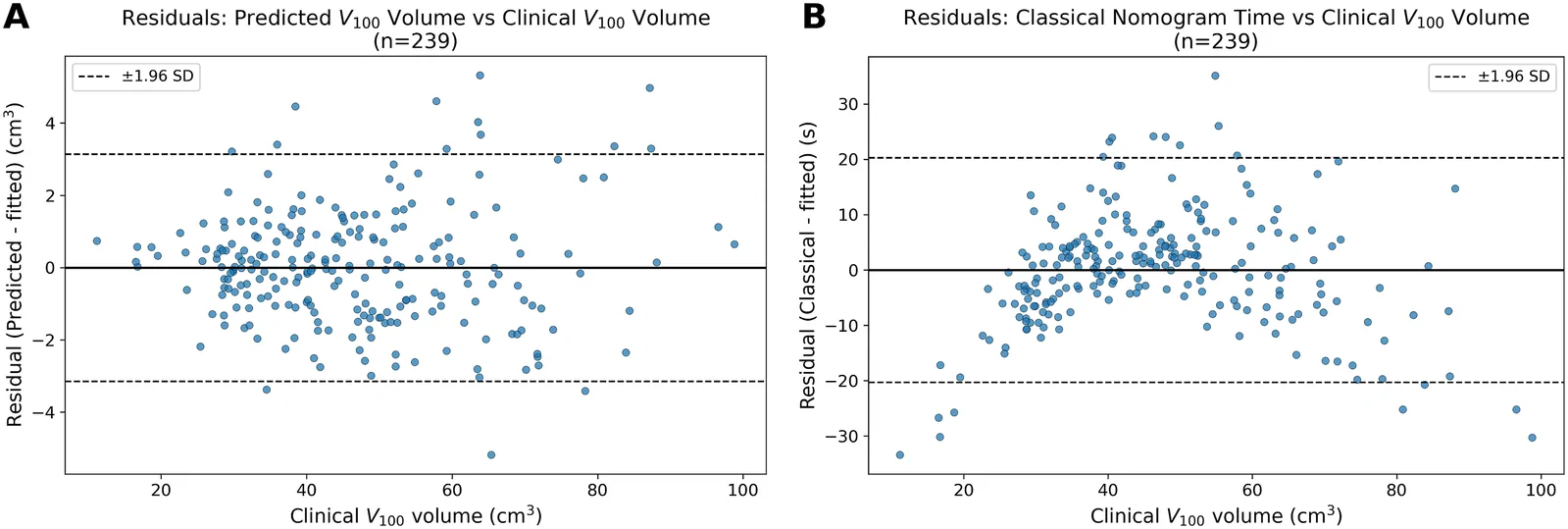
Residual analysis comparing the deep learning-based and classical nomogram approaches. (A) ${\mathrm{TV}}_{100\%}$ residuals (${\mathrm{TV}}_{100\%}^{\mathrm{pred}}$ - $TV_{100\%}^{\mathrm{clin}}$) and (B) Classical nomogram dwell-time residuals (predicted - clinical) plotted against $TV_{100\%}^{\mathrm{clin}}$. (*n* = 239).

Sensitivity testing demonstrated coherent changes in ${\mathrm{TV}}_{100\%}^{\mathrm{pred}}$ following structured perturbations of anatomy and dwell geometry in one representative case. Expansion of the prostate contour creates a scenario in which the needles, and corresponding dwells, were farther from the gland periphery, resulting in a reduced predicted peripheral coverage and a prostate $V_{100\%}^{\mathrm{pred}}$ of 92.4% (Fig. 6A). Removal of the anterior right needle eliminated dwell positions in that region, and the ${\mathrm{TV}}_{100\%}^{\mathrm{pred}}$ showed a corresponding reduction in anterior right local coverage, with prostate $V_{100\%}^{\mathrm{pred}}$ of 93.7% (Fig. 6B). Finally, relabeling urethral voxels as prostate removed the urethral structure from the input representation, and the model predicted a larger ${\mathrm{TV}}_{100\%}^{\mathrm{pred}}$ with increased prostate coverage, $V_{100\%}^{\mathrm{pred}}$ of 98.5% (Fig. 6C). These single-case perturbations illustrate that the model output responds coherently to structured changes in anatomy and dwell geometry but should not be interpreted as a comprehensive robustness evaluation or as treatment-plan optimization or dwell-time prediction.

**Fig. 6:**
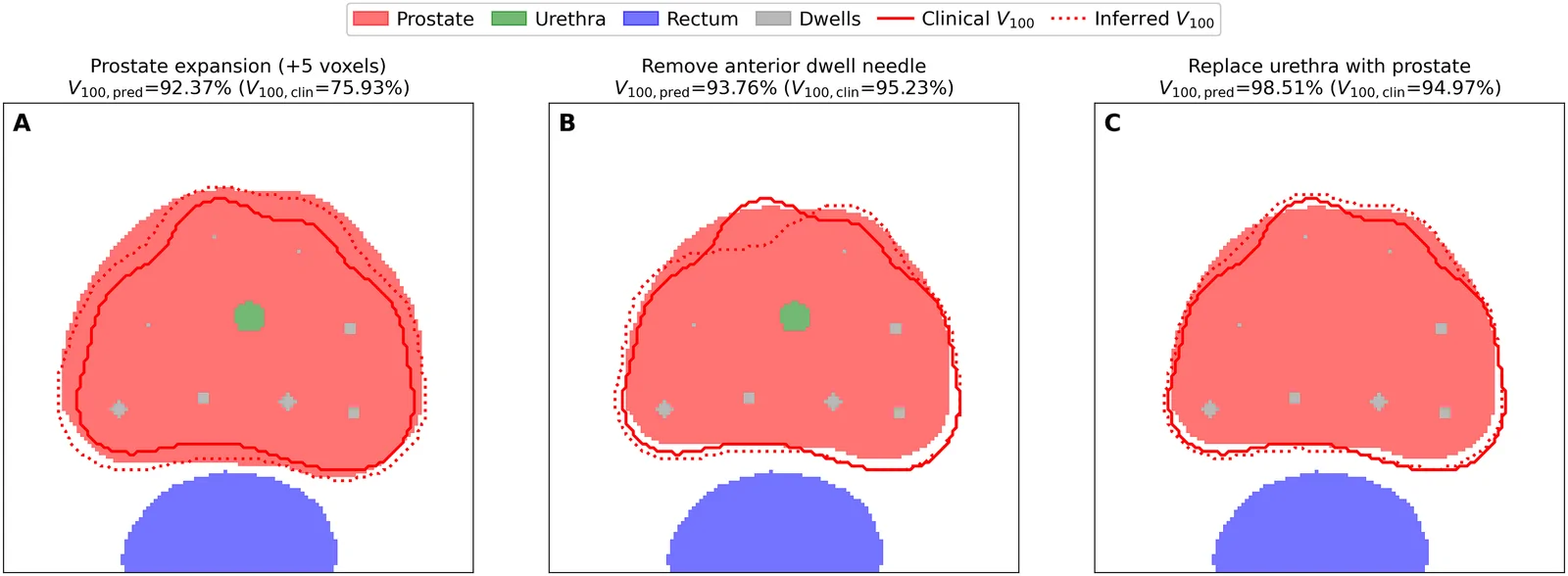
Single-case sensitivity testing (central axial slice displayed) showing three structured input perturbations: prostate expansion (+5 voxels), removal of a single anterior needle's dwell pattern (all dwell voxels along that needle track removed across slices), and replacement of the urethra contour with prostate. Each panel displays the baseline $TV_{100\%}^{\mathrm{clin}}$ (red line) and the deep-learning model's shows $TV_{100\%}^{\mathrm{pred}}$(dotted red line). (For interpretation of the references to colour in this figure legend, the reader is referred to the web version of this article.)

## 4 Discussion

This study demonstrated that deep learning-based nomograms can predict the clinical 100% isodose volume in three dimensions from segmented anatomy and planned dwell locations. Unlike classical nomograms, which operate on scalar values, the model generates a spatially resolved expectation of coverage that can be compared directly with the clinical plan using volumetric and 3D spatial metrics. This transforms independent verification from a one-number consistency check into a 3D quantitative assessment. The inclusion of dwell location is highlighted given how sensitive metrics such as prostate V_100%_ or organ-at-risk doses are to catheter reconstruction [17].

In a proposed clinical workflow, the model would be applied after plan optimization and before final plan approval and treatment delivery. Validation-derived thresholds would identify coverage patterns atypical of prior plans with similar anatomy and dwell geometry. The physicist would review agreement metrics and the three-dimensional comparison of predicted and clinical 100% isodose volumes. A threshold exceedance would prompt targeted review, not automatic rejection, and plans would be modified only if review identified a correctable discrepancy, whereas intentional deviations could be documented and accepted.

A flag would not identify a specific error type. It could directly reflect unusual optimization or indirectly indicate catheter- or dwell-reconstruction errors or contouring inconsistencies that caused the clinical isodose volume to deviate from the learned expectation.

Classical brachytherapy nomograms remain useful because they are simple, transparent, and fast. However, time-based verification cannot localize discrepancies and may miss redistributions of dwell time that preserve total treatment time. The proposed deep learning-based nomogram addresses this limitation by predicting the full ${\mathrm{TV}}_{100\%}$ volume, enabling volumetric and spatial agreement checks that better reflect the achieved implant geometry. As brachytherapy delivery is sensitive to multiple sources of uncertainty, advances in independent verifications are recommended to ensure consistency between planned and delivered dose distributions [18]. This method has the potential to complement equation-based nomograms for post-plan QA by adding spatially resolved verification [7], [8], [18].

Recent learning-based work in HDR prostate brachytherapy has included 3D dose prediction, including rapid dose calculation and dose map predictions [10], [19]. Related non-prostate work has also demonstrated knowledge-based voxel-wise dose prediction for cervical tandem-and-ovoid brachytherapy [11]. Other work has focused on needle placement, plan generation, and two-dimensional prediction of isodose contours or plan-quality surrogates [20], [21], [22], [23], [24]. The present work differs as it is a 3D spatial post-plan QA nomogram for HDR prostate brachytherapy. Specifically, the model predicts ${\mathrm{TV}}_{100\%}$ from anatomy and dwell geometry and evaluates whether the resulting coverage pattern is consistent with prior institutional practice. This framing leverages the completed implant geometry as an input for independent plausibility assessment, rather than for prospective plan generation or dose calculation.

Cases flagged for review may reflect challenging anatomy, expected variation, or intentional departures from historical practice. This is particularly relevant for plans that include dominant intraprostatic lesion boosting: such cases may be appropriately flagged because they differ from the patterns represented in the training set, and they may ultimately need to be modeled separately or excluded from future training cohorts if the intended QA target is conventional whole-gland planning. Viewed this way, the model is not enforcing a single optimal plan, but rather highlighting cases in which the achieved coverage appears atypical.

Several limitations should be considered. The reference standard was the clinical ${\mathrm{TV}}_{100\%}$ volume, generated from the clinical treatment planning system dose distribution, rather than an independently recalculated or optimized physical dose distribution. The model therefore learns historical clinical planning patterns, including the combined effects of contouring practice, optimization strategy, physician preference, TPS implementation, and any systematic export or reconstruction artifacts. As a result, the proposed framework should be interpreted as a learned spatial consistency check against prior institutional plans, not as an independent TG-43 dose recalculation. This distinction is important because the model may reproduce institution-specific biases or planning-style preferences present in the training data. External validation is therefore required before broader use. The model was developed exclusively using single-fraction 15 Gy whole-gland plans and should not be assumed to generalize to focal treatments, salvage brachytherapy, or other fractionation schedules [25]. These scenarios and centres with different catheter geometries, optimization strategies, contouring practices, or TPS implementations may have different expected 100% isodose patterns and model thresholds, requiring dedicated validation and potentially recalibration or retraining.

Although agreement was strong across test-set isodose volumes, performance was not stratified by prostate size, anatomic asymmetry, or catheter configuration. Extreme or uncommon cases may be underrepresented and warrant additional physicist scrutiny. In addition, the input representation is contour-based rather than image-based: this improves robustness to image-quality variation and makes the model image-modality agnostic, but it also makes performance dependent on contour and dwell reconstruction consistency. Finally, while prostate $V_{100\%}$ was a natural primary QA endpoint for this study, other dosimetric endpoints may require separate models and may be less suitable as primary QA triggers because they can vary intentionally across cases.

Future work should evaluate prospective integration and multi-institution generalizability, including procedures for commissioning, recalibration, and monitoring for shifts in planning practice. Extensions to additional isodose levels or selected DVH endpoints may further broaden the QA utility of the approach, provided the central goal remains the same: identifying plans whose three-dimensional coverage pattern deviates from the expected behavior for a given completed implant geometry.

Overall, this study demonstrated that a deep learning-based nomogram can predict the three-dimensional 100% prescription isodose volume from anatomy and dwell geometry within an institution-specific planning framework. Validation-derived thresholds may help identify atypical plans for physicist review alongside, but not in place of, established HDR quality-assurance procedures. External validation is required before broader clinical implementation.

## CRediT authorship contribution statement

**Joel Beaudry:** Writing – original draft, Visualization, Validation, Software, Resources, Project administration, Methodology, Investigation, Formal analysis, Data curation, Conceptualization. **Tonghe Wang:** Writing – review & editing, Methodology. **Shirin A. Enger:** Writing – review & editing, Supervision, Methodology, Investigation, Conceptualization. **Antonio L. Damato:** Writing – review & editing, Supervision, Resources, Project administration, Methodology, Investigation, Conceptualization.

## Ethics declaration

Written informed consent to take part in the study and to publish the article has been obtained from all participants or their legal representatives. The privacy rights of participants have been observed.

This study was performed in compliance with relevant laws, regulatory frameworks and guidelines where the research took place.

## Declaration of competing interest

The authors declare that they have no known competing financial interests or personal relationships that could have appeared to influence the work reported in this paper.

## Appendix A Supplementary data

Supplementary material AI-RT reporting card for the deep learning–based HDR prostate brachytherapy nomogram.
