# Supplementary material for: Three-dimensional deep learning nomogram for post-plan quality assurance in high-dose-rate prostate brachytherapy
Source: Phys Imaging Radiat Oncol. 2026 Oct 2;41:101100. doi: 10.1016/j.phro.2026.101100 (PMC13679370; doi:10.1016/j.phro.2026.101100)
Supplement: Supplementary file 1 [file mmc1.pdf]

Model Card — Model Three-dimensional deep learning nomogram for HDR prostate brachytherapy QA

Task: Segmentation / Binary Segmentation

0. Card Metadata

**Creation date * ** : 2026/08/17

Versioning

- **Version number * ** : 1.0
- **Version changes * ** : Initial AID-RT model card

1. Model Basic Information

**Name * ** : Three-dimensional deep learning nomogram for HDR prostate brachytherapy QA

**Creation date * ** : 2025/12/12

Versioning

- **Version number * ** : 01.00.0000
- **Version changes * ** : Initial research model described in the associated manuscript

Model scope

- **Summary * ** : A manually configured three-dimensional U-Net predicts an institution-specific binary 100% prescription isodose volume from prostate, urethra, rectum, and reconstructed catheter dwell-position geometry for post-plan HDR prostate brachytherapy QA review.
- **Anatomical site * ** : Pelvis

Clearance

- **Type * ** : Not approved or cleared for medical use; retrospective research model only.

Approved by

- **Name(s)**: —
- **Institution(s) * ** : Memorial Sloan Kettering Cancer Center
- **Contact email(s)**: —

Intended users: Qualified medical physicists and the treating HDR brachytherapy team.

**Observed limitations * ** : Development and evaluation used retrospective, single-institution, single-fraction 15 Gy whole-gland HDR prostate plans. The model was evaluated as a spatial consistency check against prior approved

plans, not as an independent TG-43 dose recalculation or a validated detector of clinically meaningful errors. Dwell times were not model inputs. No external or prospective validation was performed.

Potential limitations: Performance may differ for extreme prostate sizes, highly asymmetric anatomy, uncommon catheter configurations, focal treatment, salvage brachytherapy, other prescriptions or fractionation schedules, and centres with different catheter geometry, contouring, optimization, planning, or TPS practices. Dedicated validation and potentially recalibration or retraining would be required.

**Type of learning architecture * ** : Manually configured MONAI/PyTorch 3D U-Net

Developed by

- **Name**: —
- **Institution(s) * ** : Memorial Sloan Kettering Cancer Center
- **Contact email(s)**: —

**Conflict of interest * ** : NA

**Software licence * ** : NA

---

2. Technical specifications

2.1 Model overview

Model pipeline

- **Summary * ** : TPS-derived anatomical contours and all possible dwell positions along each reconstructed catheter are converted to a categorical 3D label map, cropped and resampled, passed through each of five fold-specific 3D U-Nets, and ensemble-averaged to produce a binary expected 100% prescription isodose volume. The predicted and clinical masks are then compared using volumetric and spatial metrics for physicist review.
- **Model inputs * ** : ['Contour label maps', 'reconstructed dwell positions']
- **Additional information**: Single-channel categorical label map: 0 background, 1 prostate, 2 urethra, 3 rectum, and 4 dwell positions. All possible dwell positions along the reconstructed catheter were included; dwell times were excluded. Dwell positions were represented by a 1 mm spherical dilation.
- **Model outputs * ** : ['Binary 100% prescription isodose mask']
- **Additional information**: Binary three-dimensional mask of the predicted 100% prescription isodose volume on the model grid.
- **Pre-processing * ** : Anatomical contours and reconstructed catheter dwell positions were rasterized into a single categorical label map. Dwell positions were spherically dilated by 1 mm; dwell labels overwrote anatomical labels at overlap. Volumes were resampled to 1.0 x 0.5 x 0.5 mm and cropped to 80 x 128 x 128 voxels, centered on the origin at the top of the ultrasound probe.
- **Post-processing * ** : For each case, sigmoid probability maps from the five fold-specific models were averaged voxelwise and thresholded at 0.5. No additional morphological post-processing was applied

2.2 Learning architecture(s)

Learning architecture 1

Field	Value
**Total number of trainable parameters ***	4738058
**Number of inputs**	1
**Input content**	['Categorical anatomy and dwell-position label map']
**Additional information regarding input content**	background, prostate, urethra, rectum, dwell positions
**Input format**	Single-channel 3D tensor
**Input size**	[1,80,128,128]
**Number of outputs**	1
**Output content**	['100% prescription isodose probability map']
**Additional information regarding output content**	Converted to a binary mask after five-model ensemble averaging
**Output format**	Single-channel sigmoid probability tensor
**Output size**	[1,80,128,128]
**Loss function ***	DiceLoss(sigmoid=True) + BCEWithLogitsLoss, equal weighting
**Batch size**	4
**Regularisation**	AdamW optimizer; on-the-fly spatial augmentation; no configured dropout, mixed precision, gradient accumulation, label smoothing, or gradient clipping
**Architecture figure ***	ArchitectureDetailed.png

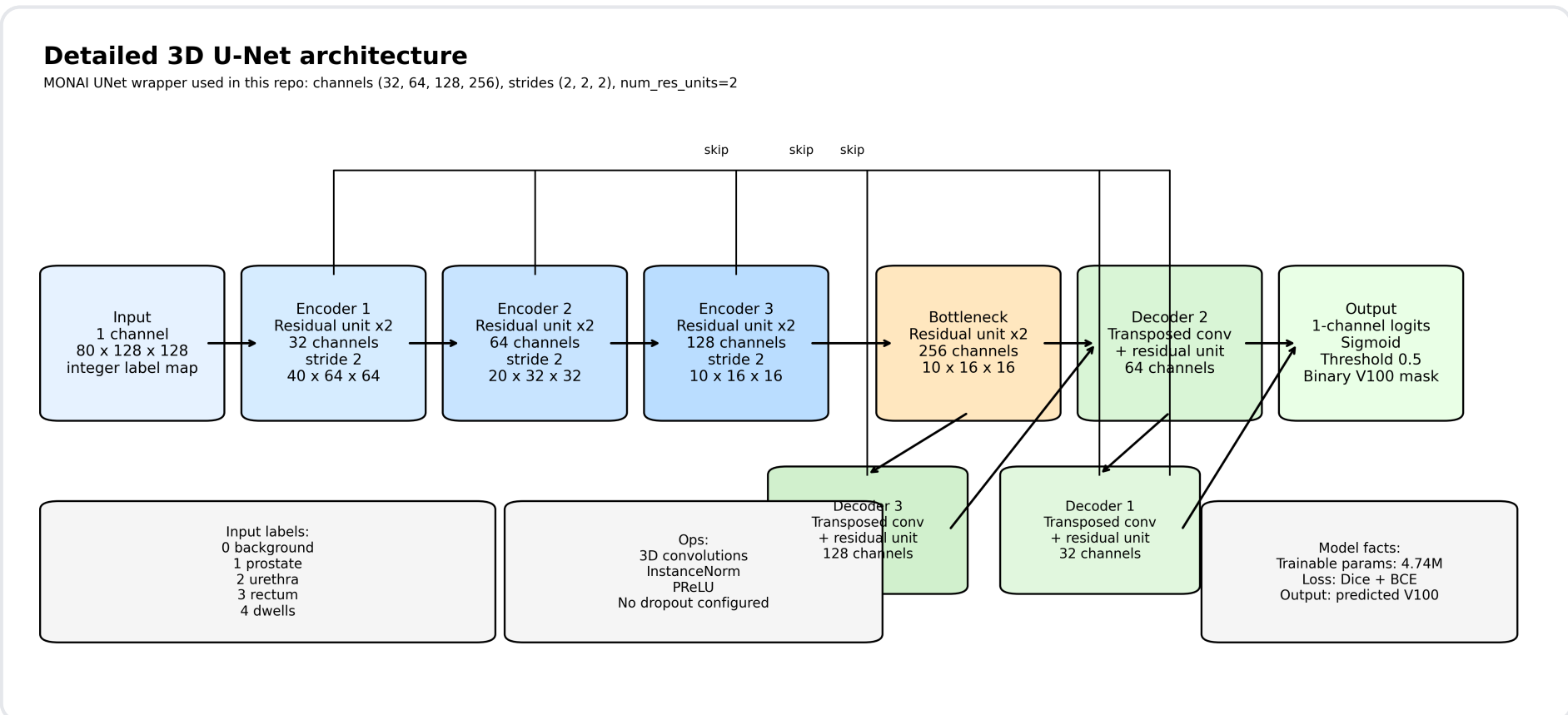

| | **Uncertainty quantification techniques *** | NA | | **Explainability techniques *** | NA | | **Additional information** | 3D UNet with channels 32/64/128/256; three stride-2 downsampling stages; 3 x 3 x 3 kernels; instance normalization; PReLU activations; transposed-convolution upsampling; skip connections; two residual units per block; no dropout. |

2.3 Hardware & software

- **Libraries and dependencies:** Python 3.12.3; PyTorch 2.6.0 (CUDA 12.4 build); torchvision 0.21.0; MONAI 1.4.0.
- **Hardware (recommended):** NVIDIA RTX 4500 GPU
- **Inference time (s) for recommended hardware:** 1.07
- **Environmental impact:** Not assessed.

---

3. Training Data Methodology and Information

Fine tuned form

- **Model name *** : NA
- **URL/DOI to model card *** : NA
- **Tuning technique *** : NA

Training Dataset

General information

- **Total size *** : [2155]
- **Number of patients *** : 2155
- **Source *** : Single-institution retrospective dataset of final approved HDR prostate brachytherapy plans.
- **Acquisition period *** : 2018-2025
- **Inclusion / exclusion criteria *** : All eligible single-fraction 15 Gy whole-gland HDR prostate plans were included. No focal treatment plans were included. One plan was included per patient.
- **Type of data augmentation**: Random left-right and superior-inferior flips (probability 0.5 each) and random translation up to 10, 20, and 20 voxels by axis (probability 0.5), with nearest-neighbour interpolation and zero padding.
- **Strategy for data augmentation:** [Random, on-the-fly during training]

Technical specifications

Contour label maps (model_inputs)

Field	Value
**Image resolution ***	[1.0, 0.5, 0.5] mm
**Patient positioning ***	NA

Field	Value
**Scan(s) manufacturer and model ***	NA
**Scan acquisition parameters ***	NA
**Scan reconstruction parameters ***	NA
**FOV ***	[64, 64, 80] mm fixed model-ready field of view (left-right, posterior-anterior, inferior-superior).

reconstructed dwell positions (model_inputs)

Field	Value
**Image resolution ***	[1.0, 0.5, 0.5] mm
**Patient positioning ***	NA
**Scan(s) manufacturer and model ***	NA
**Scan acquisition parameters ***	NA
**Scan reconstruction parameters ***	NA
**FOV ***	[64, 64, 80] mm fixed model-ready field of view (left-right, posterior-anterior, inferior-superior).

Binary 100% prescription isodose mask (model_outputs)

Field	Value
**Image resolution ***	[1.0, 0.5, 0.5] mm
**Patient positioning ***	NA
**Scan(s) manufacturer and model ***	NA
**Scan acquisition parameters ***	NA
**Scan reconstruction parameters ***	NA
**FOV ***	[64, 64, 80] mm fixed model-ready field of view (left-right, posterior-anterior, inferior-superior).

- **Reference standard *** : Binary mask generated from the final approved clinical TG-43 dose distribution at dose greater than or equal to 15 Gy (100% prescription).
- **Reference standard QA *** : Final clinically approved and treatable plan after the institution's established HDR planning and physics QA workflow
- **Additional information**: The reference plans represent final, clinically accepted institutional practice.

Patient demographics and clinical characteristics

- **Age *** : NA

- **Sex *** : Male
- **Additional patient info**: Plans were produced in Vitesse version 4.0 using ultrasound-based planning. **Validation strategy *** : 2,155 patients underwent five-fold cross-validation at the patient level.

Validation data partition * : 20% per fold (431 patients), with 80% used for training (1,724 patients) within each fold. A separate held-out test set comprised 239 patients.

Weights initialization: MONAI/PyTorch default module initialization **Epochs**: [200] **Optimiser**: AdamW. ReduceLROnPlateau scheduler monitored validation Dice with patience 3 and factor 0.5. **Learning rate**: Initial learning rate 1 x 10^-4

Model choice criteria * : Highest validation Dice within each fold.

Inference method * : Voxelwise mean of sigmoid probability maps from the five fold-specific models, followed by thresholding at 0.5

4. Evaluation Data Methodology, Results and Commissioning

1 Test Set

Evaluation date * : 2025/12/17

Evaluated by

- **Name(s) *** : Authors of the associated manuscript
- **Institution(s) *** : MSKCC
- **Contact email(s) *** : beaudrj1@mskcc.org
- **Same as 'Approved by':** No

Evaluation frame * : Retrospective, single-institution technical evaluation on a held-out test set excluded from model development. No prospective clinical commissioning or external validation was performed.

Evaluation dataset

General information

- **Total size *** : [239]
- **Number of patients *** : 239
- **Source *** : Private single-institution retrospective dataset; held-out patients from the same clinical environment as the development cohort
- **Acquisition period *** : 2018-2025
- **Inclusion / Exclusion criteria *** : Same eligibility criteria as the development cohort; one plan per patient; whole-gland single-fraction 15 Gy HDR prostate plans only
- **URL info**: —

Technical specifications

Contour label maps (model_inputs)

Field	Value
**Image resolution ***	[1.0, 0.5, 0.5] mm
**Patient positioning ***	NA
**Scan(s) manufacturer and model ***	NA
**Scan acquisition parameters ***	NA
**Scan reconstruction parameters ***	NA
**FOV ***	[64, 64, 80] mm fixed model-ready field of view (left-right, posterior-anterior, inferior-superior).

reconstructed dwell positions (model_inputs)

Field	Value
**Image resolution ***	[1.0, 0.5, 0.5] mm
**Patient positioning ***	NA
**Scan(s) manufacturer and model ***	NA
**Scan acquisition parameters ***	NA
**Scan reconstruction parameters ***	NA
**FOV ***	[64, 64, 80] mm fixed model-ready field of view (left-right, posterior-anterior, inferior-superior).

Binary 100% prescription isodose mask (model_outputs)

Field	Value
**Image resolution ***	[1.0, 0.5, 0.5] mm
**Patient positioning ***	NA
**Scan(s) manufacturer and model ***	NA
**Scan acquisition parameters ***	NA
**Scan reconstruction parameters ***	NA
**FOV ***	[64, 64, 80] mm fixed model-ready field of view (left-right, posterior-anterior, inferior-superior).

- **Reference standard *** : Binary mask generated from the final approved clinical TG-43 dose distribution at ≥15 Gy (100% prescription).
- **Reference standard QA *** : Final clinically approved and treatable plan following the established HDR planning and physics QA workflow.

Patient demographics and clinical characteristics

- **Age *** : NA

- **Sex *** : Male

Quantitative evaluation

Other Metrics

Prostate V100% absolute error

Field	Value
Type	Prostate V100% absolute error
Additional information	Absolute difference between predicted and clinical prostate V100%, reported in percentage points.
Sample Data	Held-out test set, n = 239
Mean Data	[0.937, 0.003, 3.212,0.788]
Figure Other Appendix Label	—

Prostate V100% signed error

Field	Value
Type	Prostate V100% signed error
Additional information	Predicted minus clinical prostate V100%, reported in percentage points
Sample Data	Held-out test set, n = 239
Mean Data	[0.734,-1.649,3.212,0.981]
Figure Other Appendix Label	—

Prescription isodose volume agreement

Field	Value
Type	Absolute prescription isodose volume error (cm^3)
Additional information	Agreement between predicted and clinical 100% prescription isodose volumes.
Sample Data	Held-out test set, n = 239
Mean Data	[1.272,0,5.1,1.032]
Figure Other Appendix Label	—

Dice similarity coefficient

Field	Value
Type	Dice similarity coefficient
Additional information	Spatial overlap between thresholded predicted and clinical binary 100% prescription isodose masks.
Sample Data	Held-out test set, n = 239
Mean Data	[0.958,0.925,0.982,0.010]
Figure Other Appendix Label	—

Average symmetric surface distance

Field	Value
Type	Average symmetric surface distance (mm)
Additional information	Bidirectional average surface distance between predicted and clinical binary 100% prescription isodose masks.
Sample Data	Held-out test set, n = 239
Mean Data	[0.495,0.265,0.866,0.110]
Figure Other Appendix Label	—

Qualitative evaluation

Evaluators information * : Quantitative retrospective analysis by the study authors.

Likert scoring

- **Method *** : NA
- **Results *** : NA

Turing test

- **Method *** : NA
- **Results *** : NA

Time saving

- **Method *** : NA
- **Results *** : NA

Other

- **Method *** : —
- **Results *** : —

Explainability * : The user can review the three-dimensional predicted and clinical 100% prescription isodose masks together with volumetric and spatial agreement metrics.

Citation details: —

---

5. Other considerations

- **Responsible Use and Ethical Considerations:** The model is research-only and should be used, if prospectively validated and locally commissioned, as a complementary physicist-review aid alongside and not in place of established HDR QA, independent verification, or clinical judgment.
- **Risk Analysis:** A false-negative consistency result could provide inappropriate reassurance; a false-positive result could add review burden or delay. Because the model learns prior institutional planning patterns, it may reproduce historical biases or fail under workflow, population, TPS, contouring, implant, or planning changes. A threshold exceedance should prompt targeted physicist review; a plan should be modified only when that review identifies a correctable discrepancy. Clinically intentional deviations may be documented and accepted.
- **Post-Market Surveillance / Live Monitoring:** Not applicable because the model is not deployed. Any future clinical use would require local commissioning, prospective validation, documented override and escalation procedures, monitoring of input and performance drift, periodic threshold review, incident review, version control, and revalidation after material workflow or software changes.
